# Supplementary material for: PU.1 Expression Defines Distinct Functional Activities in the Phenotypic HSC Compartment of a Murine Inflammatory Stress Model
Source: Cells. 2022 Feb 15;11(4):680. doi: 10.3390/cells11040680 (PMC8870714; doi:10.3390/cells11040680)
Supplement: Supplementary file 1 [file cells-11-00680-s001.zip › cells-1456839-supplementary.pdf]

cells MDPI

Supplementary Figure

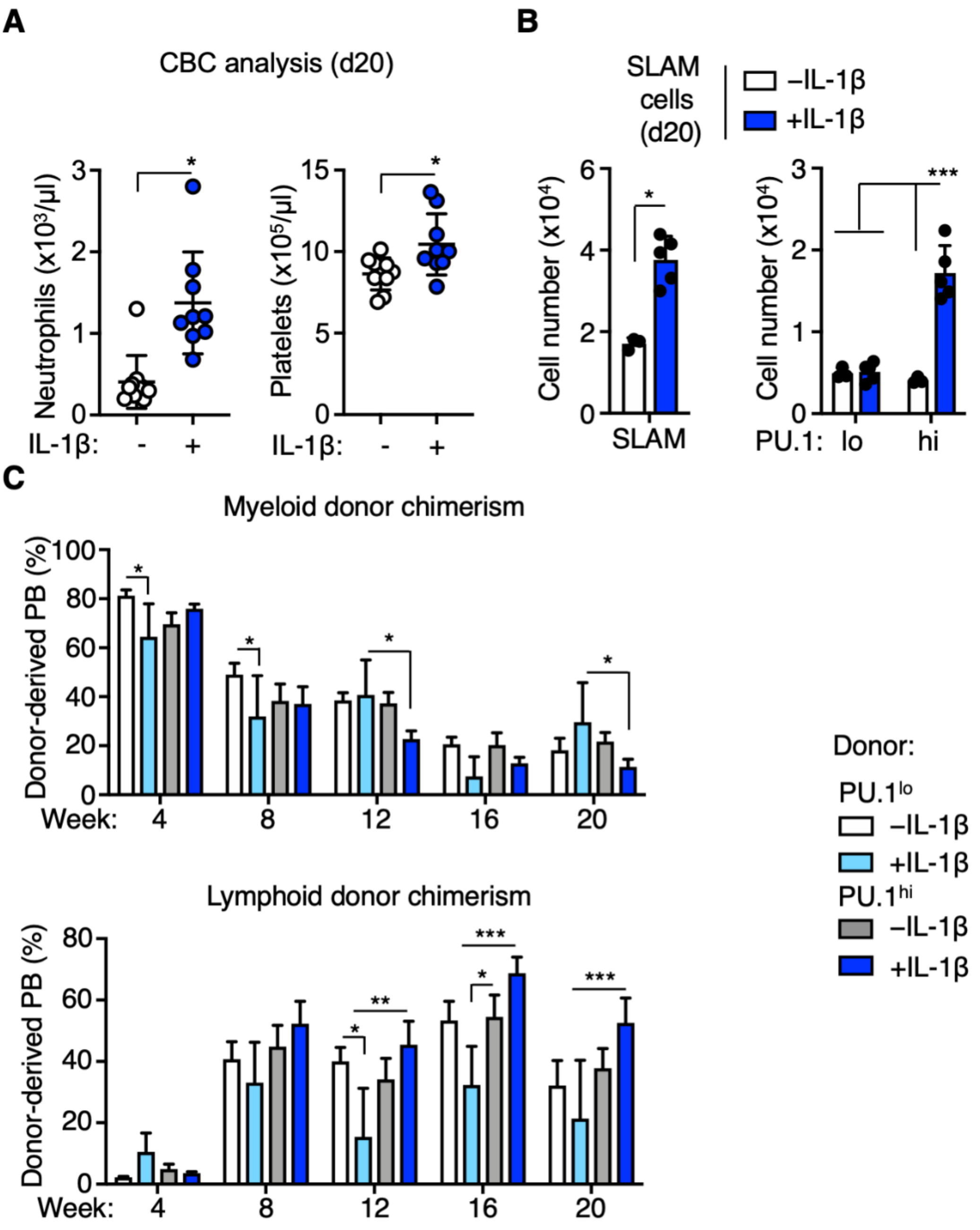

Figure S1. CBC analysis, SLAM cell absolute numbers and lineage distribution of transplant experiments. (**A**) Complete blood count (CBC) analysis of blood from mice treated for 20d ± IL-1 (n = 9/grp). Means and individual values are shown. Data are representative of two independent experiments. (**B**) Absolute numbers of total SLAM cells (left) and PU.1$^{lo/hi}$ SLAM cell fractions in the four long bones of mice treated for 20d ± IL-1 (n = 3-5/grp). Means and individual values are shown. Data are representative of at least two independent experiments. (**C**) Summary data showing donor myeloid (top) and lymphoid (bottom) lineage distribution in peripheral blood of transplanted recipient mice (n = 6-8/grp). Data are compiled from two independent experiments. Bars represent mean values. Data are shown as means ± SD. Significance was determined by ANOVA with Tukey's post-test. *p<0.05; **p<0.01; ***p<0.001.

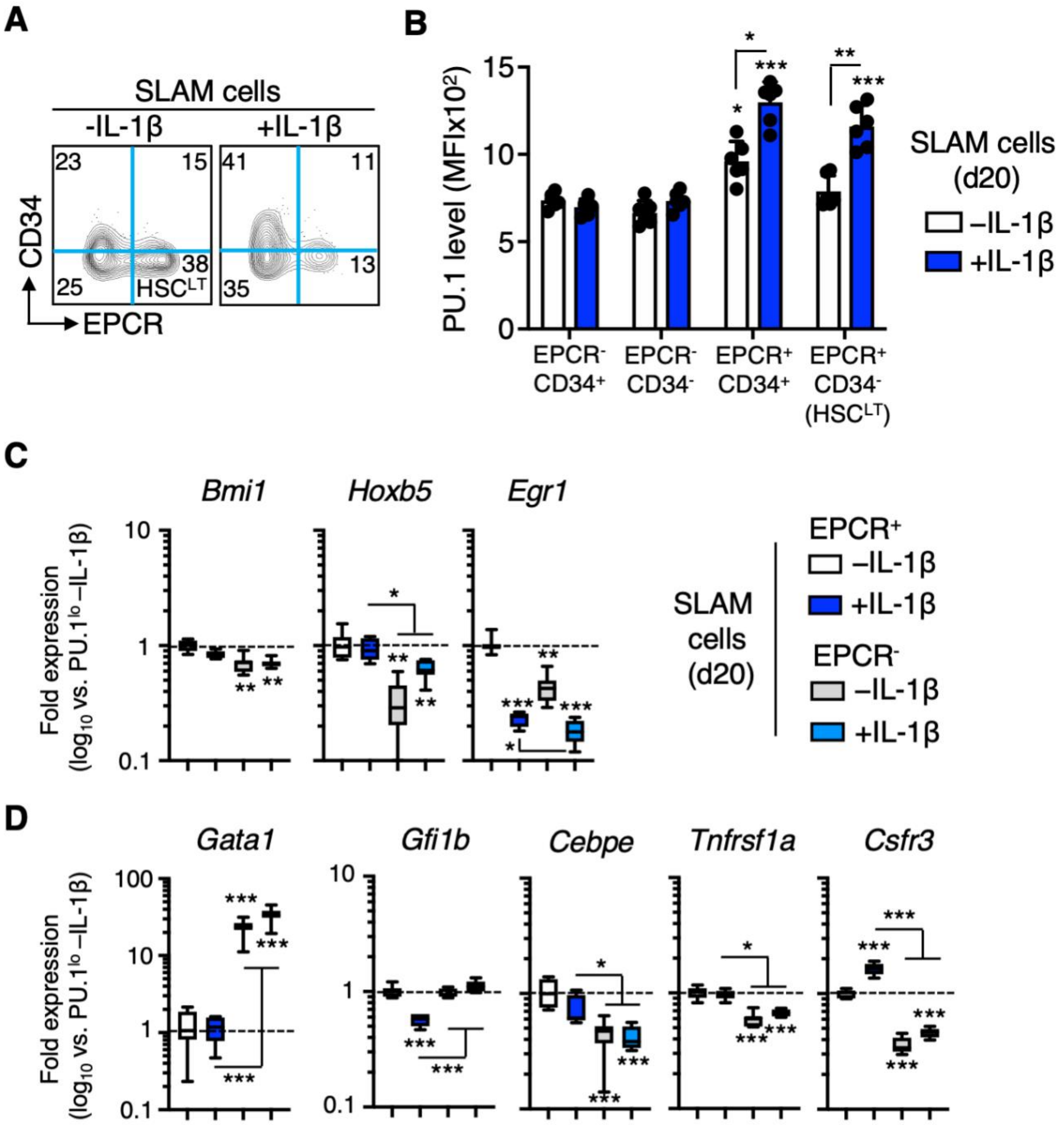

Figure S2. PU.1 levels and gene expression in EPCR SLAM cell fractions. (**A**) Representative FACS plot showing fractionation of SLAM cells using EPCR and CD34. (**B**) Analysis of *PU.1-EYFP* reporter expression in SLAM fractions from mice treated for 20d ± IL-1β (n=6/grp). Data are expressed as geometric mean fluorescence intensity (MFI). Individual values are shown, bars represent mean values. Data are representative of two independent experiments. (**C**–**D**) Fluidigm qRT-PCR analysis of (**C**) HSC genes and (**D**) megakaryocyte/erythroid and myeloid lineage genes in EPCR$^+$ and EPCR$^-$ SLAM cells from mice treated for 20d ± IL-1β (n=8/grp). Data are expressed as log_{10} fold change versus EPCR$^+$ SLAM cells from -IL-1β control mice. Box represents upper and lower quartiles with line representing median value. Whiskers represent minimum and maximum values. Data are representative of two independent experiments. Data are shown as means ± SD. Significance was determined by ANOVA with Tukey's post-test. *p<0.05; **p<0.01; ***p<0.001.

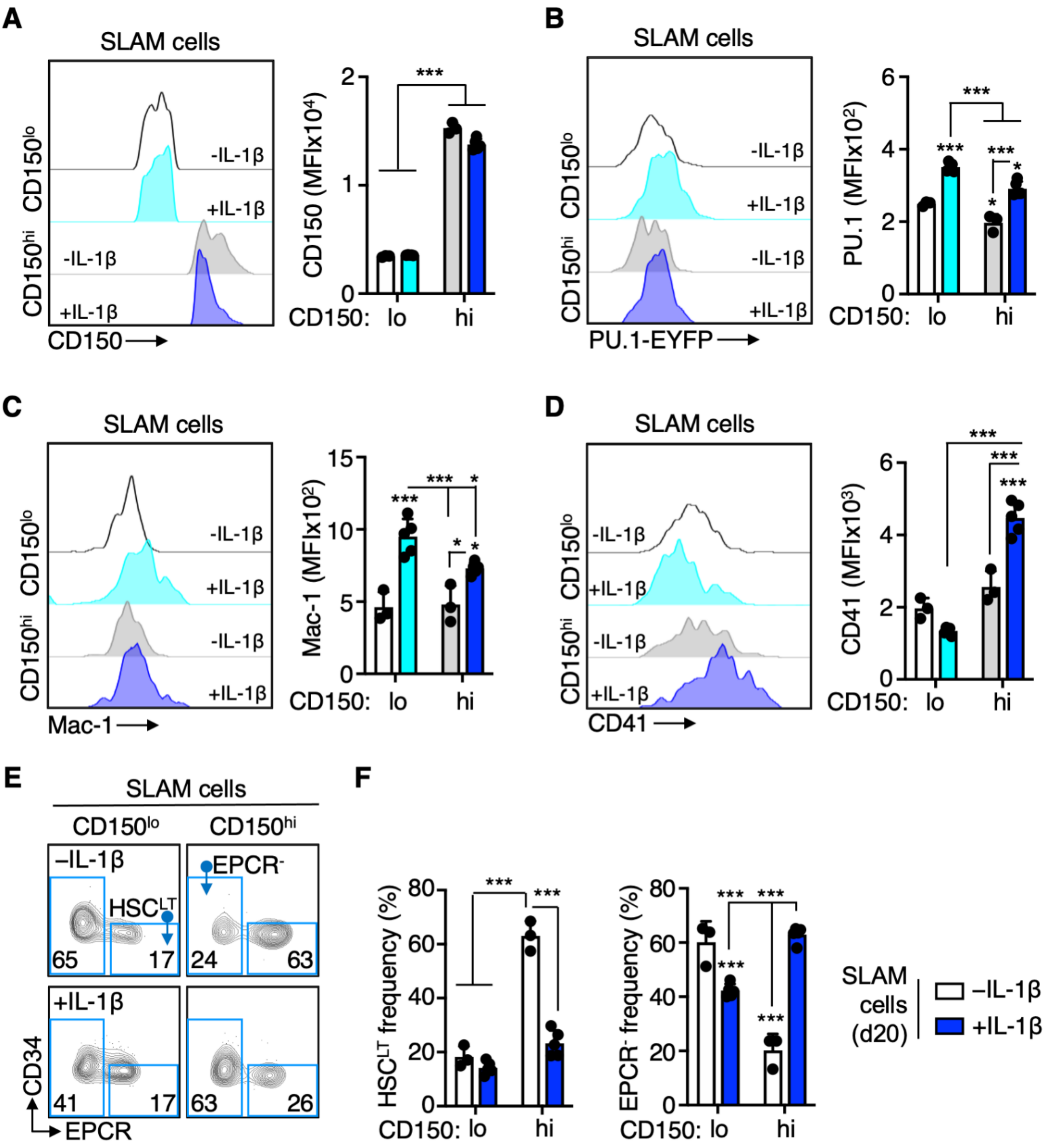

Figure S3. HSC and lineage marker expression in CD150^{lo} and CD150^{hi} SLAM fractions. (**A**) Representative histogram (left) and summary data (right) showing fractionation of SLAM cells by CD150 expression level in *PU.1-EYFP* mice treated for 20d ± IL-1β (n = 3-5/grp). Data are expressed as geometric mean fluorescence intensity (MFI). Individual values are shown, bars represent mean values. Data are representative of two independent experiments. (**B–D**) Representative histogram (left) and summary data (right) showing B) PU.1-EYFP levels, C) Mac-1 levels, and D) CD41 levels in SLAM cells by fractionated CD150 expression level from *PU.1-EYFP* mice treated for 20d ± IL-1β (n = 3-5/grp). Data are expressed as geometric mean fluorescence intensity (MFI). Individual values are shown, bars represent mean values. Data are representative of two independent experiments. (**E**) Representative FACS plot showing frequencies of HSC^{LT} and EPCR^{-} SLAM cells within the CD150^{hi} and CD150^{lo} SLAM fractions in *PU.1-EYFP* mice treated for 20d ± IL-1β (n=3-5/grp). (**F**) Summary data showing the frequencies of HSC^{LT} and EPCR^{-} SLAM cells within the CD150^{hi} and CD150^{lo} SLAM fractions in *PU.1-*

EYFP mice treated for 20d ± IL-1β (n = 3-5/grp). Individual values are shown, bars represent mean values. Data are representative of two independent experiments. Data are shown as means ± SD. Significance was determined by ANOVA with Tukey's post-test. *$p<0.05$; **$p<0.01$; ***$p<0.001$.

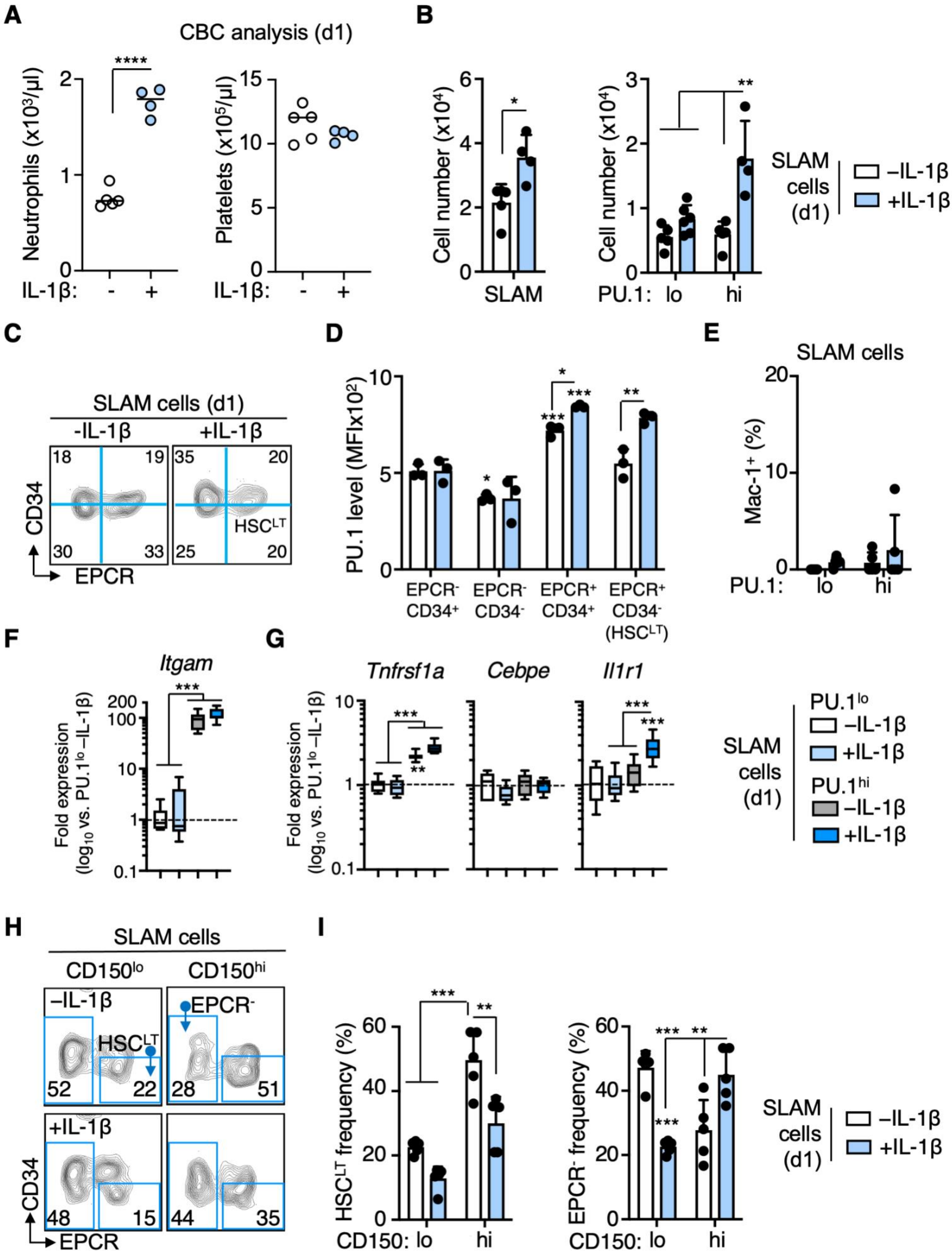

Figure S4. PU.1 levels and gene expression in SLAM cell fractions following acute IL-1β treatment. (**A**) Complete blood count (CBC) analysis of blood from mice treated for 1d ± IL-1β (n = 4-5/grp). Means and individual values are shown. Data are representative of two independent experiments. (**B**) Absolute numbers of total SLAM cells (left) and PU.1$^{lo/hi}$ SLAM

cell fractions in the four long bones of mice treated for 20d ± IL-1β (n = 4-6/grp). Means and individual values are shown. Data are representative of at least two independent experiments. (**C**) Representative FACS plot showing fractionation of SLAM cells using EPCR and CD34. (**D**) Analysis of *PU.1-EYFP* reporter expression in SLAM fractions from mice treated for 1d ± IL-1β (n = 3/grp). Data are expressed as geometric mean fluorescence intensity (MFI). Individual values are shown, bars represent mean values. Data are representative of two independent experiments. (**E**) Frequency of Mac-1+ cells within the SLAM gate of *PU.1-EYFP* mice treated for 20d ± IL-1β (n = 5/grp). Individual values are shown, bars represent mean values. Data are representative of two independent experiments. (**F–G**) Fluidigm qRT-PCR analysis of F) *Itgam* (*Mac-1*) and G) myeloid lineage genes in PU.1^{hi} and PU.1^{lo} SLAM cells from mice treated for 20d ± IL-1β (n=8/grp). Data are expressed as log_{10} fold change versus $EPCR^{+}$ SLAM cells from -IL-1β control mice. Box represents upper and lower quartiles with line representing median value. Whiskers represent minimum and maximum values. Data are representative of two independent experiments. (**H**) Representative FACS plot showing frequencies of HSC^{LT} and $EPCR^{-}$ SLAM cells within the $CD150^{hi}$ and $CD150^{lo}$ SLAM fractions in *PU.1-EYFP* mice treated for 20d ± IL-1β (n=3-5/grp). (**I**) Summary data showing the frequencies of HSC^{LT} and $EPCR^{-}$ SLAM cells within the $CD150^{hi}$ and $CD150^{lo}$ SLAM fractions in *PU.1-EYFP* mice treated for 20d ± IL-1β (n = 3-5/grp). Individual values are shown, bars represent mean values. Data are representative of two independent experiments. Data are shown as means ± SD. Significance was determined by ANOVA with Tukey's post-test. *p<0.05; **p<0.01; ***p<0.001.

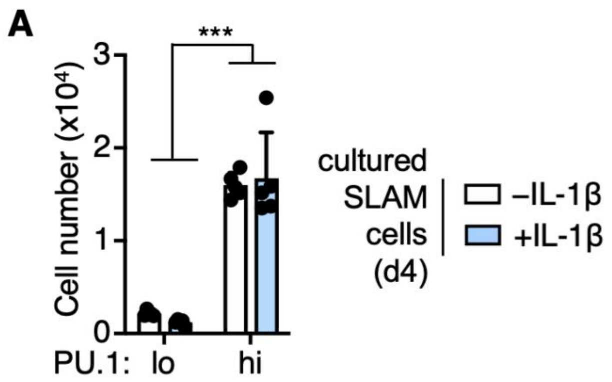

Figure S5. Growth kinetics of PU.1^{lo} and PU.1^{hi} SLAM cells in liquid culture. (**A**) Summary data showing number of cells after 4 days culture of PU.1^{lo} and PU.1^{hi} SLAM cells from *PU.1-EYFP* mice treated for 1d ± IL-1β (n = 5/grp). Mean values are shown. Data are representative of two independent experiments. Data are shown as means ± SD. Significance was determined by ANOVA with Tukey's post-test. ***p<0.001.
